# The effects of concurrent training (aerobic-resistance) and milk consumption on some markers of bone mineral density in women with osteoporosis

**DOI:** 10.1186/s12905-018-0694-x

**Published:** 2018-12-17

**Authors:** Hamid Arazi, Mahbobeh Samadpour, Ehsan Eghbali

**Affiliations:** 1 Department of Exercise Physiology, Faculty of Sport Sciences, University of Guilan, Rasht, Iran; 2 Department of Exercise Physiology, Islamic Azad University, Rasht Branch, Rasht, Iran; 3 Department of Exercise Physiology, Faculty of Sport Sciences, University of Guilan, Rasht, Iran

**Keywords:** Osteoporosis, Concurrent training, Milk, 25-Hydroxyvitamin D, Alkaline phosphatase

## Abstract

**Background:**

Osteoporosis is a skeletal metabolic disorder characterized by low bone mineral density (BMD) and reduced bone strength leading to higher bone fractures risk. The present study attempted to investigate the effects of concurrent training (aerobic-resistance) and milk consumption on some markers of BMD in women with osteoporosis.

**Methods:**

For this purpose, forty women diagnosed with osteoporosis within an age range of 30-45 years were divided into four groups of ten including concurrent training-milk, concurrent training, milk consumption and control group. The concurrent exercises were performed in ten weeks with three sessions in each week including aerobic training (running at 55–75% of maximum heart rate) and resistance training (4 move in a circle performed two times with 10 repetition maximum (RM)). Milk consumption was two times of 250 ml per day in ten weeks. Before and after treatment, BMDs in the hip and lumbar spine area were estimated with Dual-energy X-ray absorptiometry (DEXA) device and 5 cc blood was taken from a vein in the arm to determine the blood levels of 25-hydroxyvitamin D (25OH-D) and alkaline phosphatase (ALP).

**Results:**

Based on the results, blood levels 25OH-D and ALP significantly increased in concurrent training-milk, concurrent training and milk group with higher increase in concurrent training-milk group (*P* < 0.05). Furthermore, the right and left hip BMD in concurrent training-milk and concurrent training groups increased significantly with higher increase in concurrent training-milk group (*p* < 0.05). Also, lumbar spine BMD increased significantly in concurrent training-milk and concurrent training (*p* < 0.05).

**Conclusions:**

It seems that combination of concurrent training and milk consumption has more efficient impacts on the BMD of young women diagnosed with osteoporosis compared to the milk or concurrent training groups alone. This treatment can be used as an effective way to improve BMD in young women with diagnosed osteoporosis.

## Background

Osteoporosis is a skeletal systemic and metabolic disease characterized by low bone density and high fracture leading to lower bone strength and in turn, higher risk of fracture [1, 2]. Osteoporosis is recognized as the fourth dangerous disease after heart disease, cancer and diabetes by World Health Organization (WHO) [1, 2].

Development and increase of osteoporosis can be attributed to lifestyle and dimorphic factors [3]. Maintaining a favorable nutrition status is a major contributor to the prevention of osteoporosis. One of the main food groups is dairy foods, one of the richest sources of nutrients, such as protein, calcium, magnesium and B vitamins, which helps expanding bone health [4]. Many believe that milk is a very important nutritional material for increasing bone mass. It is one of the main sources of calcium and other elements necessary for the body including vitamin D, protein, potassium and phosphorus [5]. Calcium is a vital element of BMD which decreases the risk of bone fractures in people diagnosed with osteoporosis [6]. Furthermore, dairy foods can increase lean body mass and fat oxidation [7]. Among the indicators of BMD, ALP and serum levels of 25OH-D have an important role in BMD. As a biomarker of bone, ALP is an enzyme which is found in various tissues throughout the body including the liver, bone, kidney, intestine and placenta. It is mainly produced in liver and bone marrow. In bone diseases, ALP levels increase due to osteoblast activity and the amount varies depending upon age and sex [8]. Vitamin D is present in the diet as cholecalciferol. It is the only vitamin that needs a certain amount of direct sunlight besides daily diet. Vitamin D is stored in several parts of the body such as the skin and stimulates calcium absorption from the small intestine and increases calcium reabsorption from the stems and distal renal tubules [6–8].

Regular physical activity decreases the risk of health problems including obesity, heart disease, stroke, colon cancer, diabetes and osteoporosis [9]. It is considered as a preventive approach to reduce the risk of osteoporosis, falls and fractures. Physical activities such as gymnastics and weightlifting, which leads to putting a high load on the body and producing strong forces of gravity, have higher osteogenic impacts compared to activities such as swimming and cycling which consume a lot of energy [10]. Evidence suggests that greater load has more beneficial effect compared to repeated load [11].

Physical activity an important role in the development and maintenance of BMD [12]. Physical activities improve bone strength through creation of intermittent mechanical stress on the skeletal system. Research has shown that the type, intensity and duration of exercise have an influence on the BMD. So, physical activity plays an important role in the development and maintenance of bone mass and density. Peak of the force is an important and decisive factor in the analysis of mechanical load to the bone [13, 14].

Osteogenic response mechanism to load suggests that a long time physical activity has anabolic effects on the bones and improve bone density and strength. Results of the experimental research showed that a long time physical activity course with high-speed traction and high peak force had greater impact on bone osteogenic response compared to high repetition exercises with low power [15, 16]. Studies have shown that training for a long time (6-36 months) has positive effects on increasing bone density while training for a short time (less than 6 months) did not show similar results [15, 16].

Marques et al. [17] showed that eight months of resistance training may be more effective than aerobic exercise and can produce favorable changes in BMD and muscle strength. Also, the result of Hawley et al. [18] showed that resistance exercise is effective in increasing BMD in healthy young men, but has no effect on BMD in the women. Age appropriate exercises can cause balance, flexibility, coordination and muscle growth, because the muscles support the bones and also blood supply to the bones and joints increases and prevent fall and fracture.

According to the reviewed literature related to diet and exercises, it is not yet clear whether these factors can separately increase the BMD in young women with osteoporosis or they can do that more efficiently when combined together? Therefore, this study aimed at investigating the benefits of concurrent exercises including aerobic and resistance exercises (the American College of Sports Medicine in recent years compared this model to aerobic or resistance exercises alone) accompanied with milk consumption in young women with osteoporosis compared to applying them separately.

## Methods

The participants in this study included 40 Iranian young women diagnosed with osteoporosis within the age range of 30-45 years. Participants declared their preparation on a voluntary basis, after announcements in medical offices and associated laboratories belong to city of Bandar-e Anzali. The criteria for inclusion were: lack of diseases such as thyroid, diabetes, no use of certain drugs (drugs affecting BMD i.e., corticosteroids, …), sedentary lifestyle (lack of regular exercise) and not using low-fat dairy (milk, yogurt, cheese) as a source of vitamin D. Participants in the study used usual diets and received little sunlight due to their geographical and religious conditions and the type of clothing worn. Dietary intake was assessed by three-day dietary record (calcium intake was 300–650 mg per day). In addition, subjects only had daily physical activity (activities related to daily living that may include house cleaning, grocery shopping, laundry etc., but not specifically games/sports and other forms of physical activity). The University Ethics Committee approved the terms of the project.

Among the 150 persons visiting densitometry centers, 40 persons were eligible to participate in this study; they were randomly divided into four groups of ten including concurrent training-milk, concurrent training, milk consumption and control group. After making the participants aware of the research goals and procedure, they were asked to fill the consent form. Then, BMD of the lumbar spine and hip of the participants were taken by the technician. Two to three days before the start of training, with regard to the fifth day of the menstrual cycle (8-10 h fasting), blood test was taken to determine 25OH-D and ALP for the four groups (concurrent training-milk, concurrent training, milk consumption and control). The starts of training and taking blood test were adjusted with regard to the fifth day of the menstrual cycle for each woman. The pre and post tests were conducted before and after two and a half months of concurrent training and milk consumption for different groups.

The participants in the milk consumption and concurrent training-milk consumed 500 ml of daily milk for 10 weeks [14]. The milk consumption and training group and the milk consumption group drank milk immediately (250 ml) and one hour after training (250 ml). Commercial low-fat milk was used in this study. The components of this milk per 250 mL are as follows: energy, 113 kcal; protein, 8.0 g; carbohydrate, 11.5 g; fat, 3.8 g; calcium, 338 mg; zinc, 1.0 mg; iodine, 33 μg; vitamin A, 400 μg; vitamin D3, 6.25 μg; vitamin B2, 0.33 mg; and vitamin B12, 1.30 μg.

After measuring the height and weight, body mass index (BMI) was calculated by dividing weight (kg) by the square of height (m). Also, to measure waist to hip ratio (WHR), the waist circumference was divided to hip circumference. The researcher used Jackson & Pollock method to measure body fat percentage and subcutaneous fat thickness of three points of the body including the triceps, supraspinale and thigh and the body density was determined for each individual [19]. Then, using Brozek equation, body fat percentage was measured for each individual [20]. Skin fold thickness was obtained using calipers calibrated (Lafayette model o1127, USA) to an accuracy of 1 mm. All the measurement was done on the right side of the body in three times and the average number was used as the main number.

### Training protocol

**Fig. 1:**
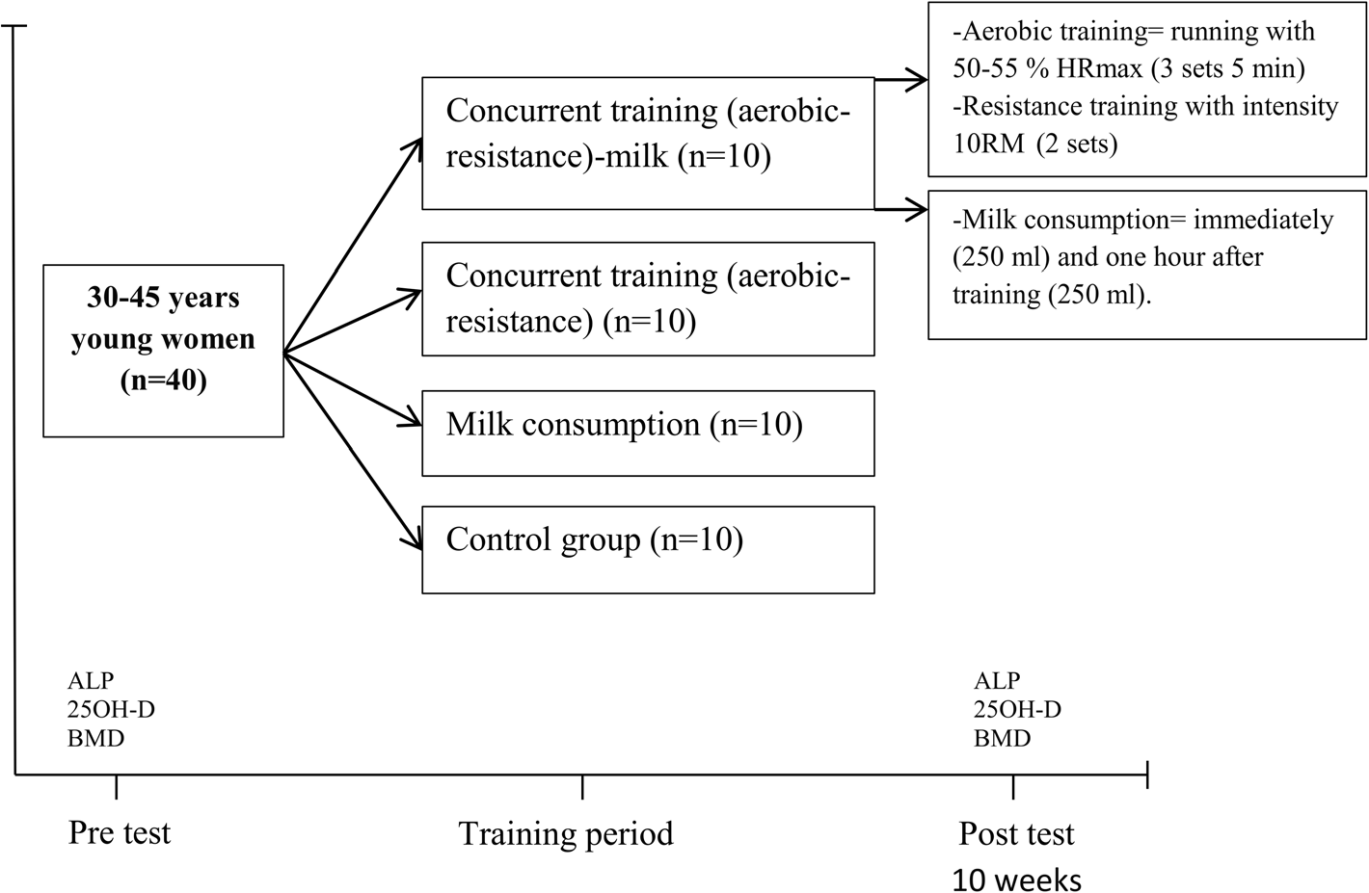
Study design. Alkaline phosphatase (ALP); 25-hydroxyvitamin D (25OH-D); bone mineral density (BMD)

The concurrent training (aerobic-resistance) was performed by groups in 10 weeks and 3 sessions each weak (each session lasted approximately 90–110 min). At the beginning, the training started with stretching and warm-up exercises (10-15 min) and continued with aerobic training (25-40 min), resistance training (working with different devices (25-40 min)) and cool-down/relaxation exercises (10-15 min). Aerobic training included 3 sets of 5 min, running with 55–75% of heart rate maximum (HRmax) of the target (heart rate during exercise was continuously monitored by heart rate monitors (Polar, AXN500)) and exercise intensity gradually increased for 5% HRmax and 3-5 min in every two weeks (rest period of approximately 3 min between each set). Resistance training involved performing 2 sets of bench press, leg extension, wide grip pull-down and leg curls which were circular with 10RM (subjects exercised on variable resistance machines). To minimize fatigue, the exercises for the upper/lower parts of the body were performed in a non-consecutive way, with a rest period of approximately 1-2 min between each set. Training intensity was gradually increased in every two weeks for new 10RM (Fig. 1).

### Blood analysis, 25OH-D and ALP

The participants were fasting 8-10 h before the experiment. Measuring blood levels of 25OH-D and serum ALP was conducted for 48 h before and after training for 5 cc from the brachial vein in sitting position (initial measurements were adjusted with regard to the fifth day of the menstrual cycle for each woman). Finally, after being transported to the laboratory and centrifuged at 3000 rpm, the units of 25OH-D and ALP levels were determined. Thus, the 25OH-D was measured with CV 2 to 3% with Elisa device (Danna model) and American parts, assembled in Iran and ALP with Hitachi device (911, Japan) with a sensitivity of 3%. The Institute of Medicine (IOM) has defined “deficiency” as a 25OH-D value below 12 ng/mL (30 nmol/L) and “sufficiency” as values above 20 ng/mL (50 nmol/L) [21].

### Measurement of BMD

BMD was measured at two areas of the lumbar spine (L2-L4) and hip by DXA device (UNIGAMMA PLUS AC 230 V 50/60 Hz 400w, USA). In this analysis, BMD at the lumbar spine was estimated from L2 to L4. The hip BMD (femoral neck) used in the analysis was estimated from the right and left side. The WHO defines osteopenia and osteoporosis based on BMD: osteopenia is BMD less than − 1 and more than − 2.5 standard deviations in relation to the mean BMD of young adults, and osteoporosis is BMD less than − 2.5 standard deviations in relation to the mean BMD of young adults [22].

### Statistical analysis

Mean and standard deviation of data were calculated using descriptive statistics. The Shapiro-Wilk test was used to check for normality test of data distribution and one-way ANOVA in the baseline analysis were used. One-way Analysis of Covariance (ANCOVA) was used to compare post-tests among the groups, because some of the pre-tests were different between the 4 groups, the effects of pre-tests were adjusted through ANCOVA. In the event of a significant F- ratio, LSD post-hoc tests were used for pairwise comparisons. Paired t-test was used to compare intergroup changes. The analyses were performed using SPSS 20 and Excel 2010. The *P* ≤ 0.05 was considered as the level of significance.

## Results

Basic features and indicators of BMD in subjects are shown in Table 1. In baseline, there were no statistical differences in age, height, weight, BMI, WHR, body fat and ALP (p>0.05). However, 25OH-D, hip and lumbar spine BMD showed significant differences (*p* < 0.05) (Table 1). The effect of pre-tests adjusted through ANCOVA.

**Table 1:** Demographic characteristics and some indicators of BMD at baseline in the subject groups

Characteristic	Mean ± SD				
	CT + MC	CT	MC	C	P
Age (years)	38.30 ± 4.76	37.30 ± 3.30	37.80 ± 4.75	38.80 ± 4.23	0.49
Height (cm)	162.34 ± 7.12	163.56 ± 5.48	163.85 ± 6.32	161.34 ± 6.21	0.32
Weight (kg)	63.95 ± 5.83	64.20 ± 7.91	62.00 ± 6.27	64.50 ± 9.16	0.48
BMI (kg/m^2^)	24.40 ± 6.26	25.13 ± 6.95	24.99 ± 2.95	25.29 ± 5.72	0.46
WHR	0.73 ± 0.04	0.75 ± 0.07	0.74 ± 0.06	0.76 ± 0.06	0.27
(%) Body fat	36.56 ± 6.30	36.62 ± 8.68	35.46 ± 4.13	37.94 ± 6.21	0.46
ALP (IU/L)	134.00 ± 32.48	135.90 ± 64.62	173.30 ± 44.90	162.80 ± 51.52	0.15
25OH-D (ng/ml)	16.50 ± 9.61	25.10 ± 11.78	24.30 ± 18.05	15.70 ± 5.49	0.03
Right hip BMD (T-score)	− 1.07 ± 0.43	−0.57 ± 0.49	− 1.25 ± 0.58	−1.18 ± 0.54	0.001
Left hip BMD (T-score)	−2.56 ± 0.18	− 2.72 ± 0.18	−2.72 ± 0.20	−2.42 ± 0.28	0.01
Lumbar spine (L2-L4) BMD (T-score)	0.31 ± 0.81-	0.48 ± 0.88-	0.32 ± 0.81-	0.11 ± 0.69-	0.01

Standard deviation (SD); body mass index (BMI); waist to hip ratio (WHR); alkaline phosphatase (ALP); 25-hydroxyvitamin D (25OH-D); bone mineral density (BMD); concurrent training-milk consumption (CT + MC); concurrent training (CT); milk consumption (MC); Control (C)

**Fig. 2:**
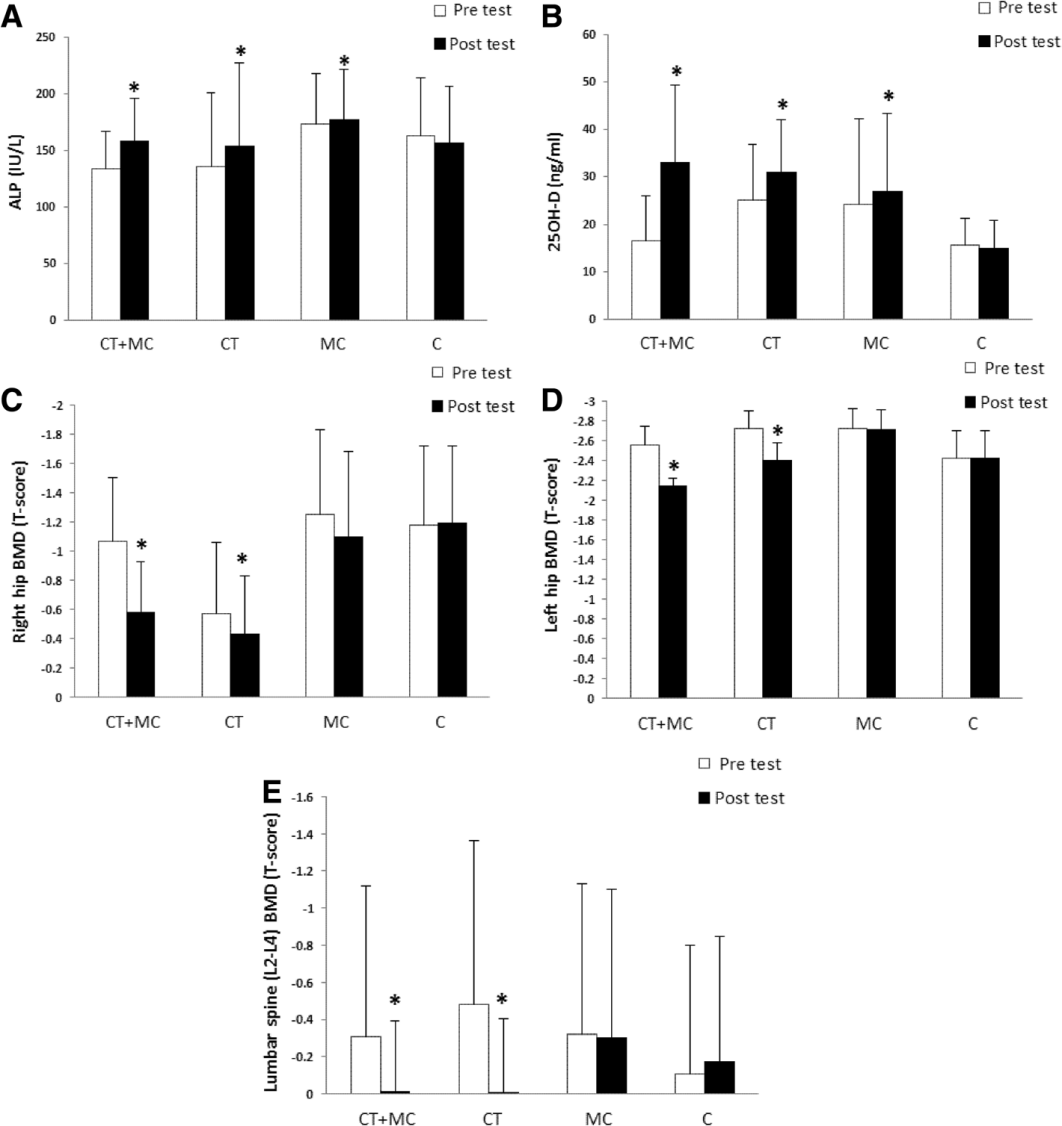
Changes in ALP (**a**), 25OH-D (**b**), right hip BMD (**c**), left hip BMD (**d**) and lumbar spine (**e**) following 10 weeks training intervention (mean ± SD). *: denotes significant differences between baseline and post training values (*p* ≤ 0.05). Standard deviation (SD); alkaline phosphatase (ALP); 25-hydroxyvitamin D (25OH-D); bone mineral density (BMD); concurrent training-milk consumption (CT + MC); concurrent training (CT); milk consumption (MC); Control (C)

Paired t test results showed that changes in 25OH-D and ALP of concurrent training-milk, concurrent training and milk consumer group were significant (25OH-D: *p* < 0.001, *p* < 0.001, *p* = 0.03; ALP: *p* < 0.001, *p* < 0.001, *p* = 0.01; respectively). Also, the changes in the hip BMD values (right and left) and lumbar spine BMD in concurrent training-milk and concurrent training were significant (right: *p* < 0.001, p = 0.01; left: *p* < 0.001, *p* < 0.001; lumbar spine: *p* = 0.02, *p* < 0.001; respectively), while there was no significant change in the milk consumer group (*p* = 0.15, *p* = 0.09, *p* = 0.10; respectively) (Fig. 2).

As demonstrated in Table 2, results of ANCOVA and LSD post-hoc test showed that there were significant differences in 25OH-D, right hip and lumbar spine BMD in the concurrent training-milk group compared to concurrent training, milk consumers and control groups (25OH-D: *p* = 0.04, *p* = 0.009, *p* < 0.001; right hip: *p* = 0.001, *p* = 0.001, p < 0.001; lumbar spine: *p* = 0.01, *p* < 0.001, *p* < 0.001; respectively). Also, 25OH-D changes in concurrent training group was significant compared to control group while there was not significant compared to the milk consumer group (*p* = 0.01, *p* = 0.49; respectively). Furthermore, ALP changes in concurrent training-milk group was significant compared to control groups (*p* = 0.03), while there was not significant compared to the concurrent training and milk consumers group (*p* = 0.73, *p* = 0.14; respectively). Changes in BMD of the left hip in concurrent training-milk group was significant compared to milk consumers and control groups (*p* < 0.001, *p* < 0.001; respectively), while there was not significant compared to the concurrent training group (*p* = 0.11). Moreover, left hip and lumbar spine BMD changes in concurrent training group were significant compared to milk consumers and control group (left hip: *p* < 0.001, *p* = 0.006; lumbar spine: *p* < 0.001, *p *< 0.001; respectively); while, changes in the right hip BMD and ALP between concurrent training with milk consumer and control groups were not significant (right hip: *p* = 0.73, *p* = 0.20; ALP: *p* = 0.24, *p* = 0.07; respectively) (Table 2).

**Table 2:** Results of ANCOVA and post hoc test

Characteristic	Groups		Mean difference	Standard error	*P*
ALP (IU/L)	CT + MC	CT	4.85	14.33	0.73
		MC	22.69	15.03	0.14
		C	32.27	14.71	0.03*
	CT	MC	17.83	15.00	0.24
		C	27.41	14.69	0.07
25OH-D (ng/ml)	CT + MC	CT	8.17	3.94	0.04*
		MC	10.75	3.86	0.009*
		C	18.10	3.70	0.001*<
	CT	MC	2.58	3.70	0.49
		C	9.93	4.00	0.01*
Right hip BMD (T-score)	CT + MC	CT	0.36	0.09	0.001*
		MC	0.33	0.09	0.001*
		C	0.50	0.08	0.001*<
	CT	MC	−0.03	0.10	0.73
		C	0.13	0.10	0.20
Left hip BMD (T-score)	CT + MC	CT	0.13	0.08	0.11
		MC	0.44	0.08	0.001*<
		C	0.38	0.08	0.001*<
	CT	MC	0.30	0.08	0.001*<
		C	0.25	0.08	0.006*
Lumbar spine (L2-L4) BMD (T-score)	CT + MC	CT	−0.12	0.05	0.01*
		MC	0.29	0.04	0.001*<
		C	0.31	0.05	0.001*<
	CT	MC	0.41	0.05	0.001*<
		C	0.44	0.06	0.001*<

Alkaline phosphatase (ALP); 25-hydroxyvitamin D (25OH-D); bone mineral density (BMD); concurrent training-milk consumption (CT + MC); concurrent training (CT); milk consumption (MC); Control (C). * denotes significant differences between groups at post training (*p* ≤ 0.05)

## Discussion

This study examined the effects of concurrent training and milk consumption on some indices of BMD in women with osteoporosis. The results showed that concurrent training and milk combination had a greater impact on BMD in young women with osteoporosis than the mere consumption of milk or concurrent training.

The ground reaction force and strain force caused by various muscle contractions during exercise can cause a certain amount of stimulation for the bone, and these mechanical stresses can improve the strength and biochemical properties of the bone. Proper mechanical stresses can cause bone formation, increase BMD, and prevent development of osteoporosis [23]. The results of the review conducted by Yuan et al. [23] showed that exercise improves BMD, bone mass, bone strength and bone mechanical properties. Physical activity seems to directly and indirectly affect all bone cells and affects many aspects of bone remodeling. Evidences suggest that exercise induces bone formation by stimulating mesenchymal stem cells, osteogenic differentiation and osteoblast and osteocyte activities; also, the exercise mechanical load and the Wnt-Catenin and the bone morphogenetic proteins pathway play an important role. In addition, the results indicated an increase in osteogenic differentiation and bone formation due to changes in levels of hormones such as parathyroid hormone (PTH), estrogen and prostaglandin E2 in exercise. Of course, the intensity and type of exercise can have different effects on individuals [23].

A few studies have investigated the impact of concurrent training on BMD while the main focus has been on investigating aerobic and resistance activities separately. The effect of separate aerobic exercise on BMD in adults is weak. The evidence for the effectiveness of this type of training to deal with age-related bone loss has been controversial [24, 25]. The results of Silverman et al. [26] showed that the bone density in femoral neck increased by 2% in postmenopausal women after 24 weeks of training in the form of walking. Aerobic training protocol usually includes walking without lateral and twisting movements which is not enough for bone density. In the ideal condition, running and walking can be effective in limiting the bone density reduction in elderly; anyway, training with average to high intensity is controversial. Low intensity aerobic training such as walking put lower pressure on the body compared to running; also, it may lead to weak stimulation of osteogenic [27]. Alghadir et al. [28] suggested that moderate-intensity aerobic exercises may protect bone and cartilage by regulating the body trace elements that are involved in the bone matrix biosynthesis and inhibit the bone resorption process through the proposed anti-free radical mechanism. Also, a study by Wen et al. [29] stated that short-term group-based step aerobics exercises are beneficial for bone metabolism and reduces bone resorption activity.

Regarding the effects of resistance training, the results of Mosti et al. [30] showed that after 12 weeks of strength training in postmenopausal women diagnosed with osteopenia and osteoporosis, 1RM and the rate of force development training group improved and the bone density in the lumbar spine and femoral neck increased for 2.9 and 4.9%, respectively. The serum type 1 collagen amino-terminal propeptide (P1NP)/type 1 collagen C breakdown products (CTX) tend to increase but not significantly, which represents stimulate bone formation. The results of Petersen et al. [31] suggested that a low-load strength exercise program with high repetitions can be an effective way to improve bone mass in adults. In addition, Watson et al. [32] suggested increased bone strength indices in postmenopausal women with low bone mass after high-intensity resistance exercise and impact training.

Contrary to these studies, the results of Almstedt et al. [18] showed that 24 weeks of resistance training is effective in increasing BMD in healthy young men (2. 7-7.7% increase), but for women, the training program did not show a significant increase in BMD (changes in BMD 0. 8-1.5%). Singh et al. [33] in his premenopausal study found that strength training in a training course of more than 9 months did not lead to significant changes in the total body BMD. Nakata et al. [34] concluded that resistance training during weight loss had no effect on BMD in premenopausal women with overweight.

Considering the dissimilarity of women responses in different ages (probably due to the deterioration of the ability of bone cells to receive physical signals or inability to respond) and the fact that estrogen deprivation is associated with increased severity of reconstruction [35], skeletal response to training is related to age. On the whole, the effect of mechanical loading forces on osteogenic reduces with aging and it seems that there is a gradual reduction of bone sensitivity to chemical and physical signals [36].

The results showed positive effects of combined concurrent training and milk consumption on BMD in young women with osteoporosis compared to consumption of milk or concurrent training, separately. In line with that, we can refer to Lester et al. [37] study which examined the effect of 8 weeks of aerobic, resistance and concurrent training on the bone biomarkers responses in inactive young women. Their results showed that the effect of osteogenic activity indices on biomarkers of bone formation (bone-specific ALP and osteocalcin) increased in the resistance and combination group. However, biomarkers of bone resorption (tartrate-resistant acid phosphatase and deoxypyridinoline) have declined in the resistance group and increased in the combination group after exercise. In the aerobic and combination groups little changes in volume and BMD were observed [37]. Moreover, Marques et al. [17] investigated the effects of aerobic and resistance training and the results showed that after eight months of training (three sessions per week), only resistance group demonstrated an increase in BMD in the trochanter (2.9%) and total hip (1.5%). They stated that both aerobic and resistance groups improved their balance but there were no significant changes in serum osteoprotegerin (OPG) and receptor activator of nuclear factor kappa B ligand (RANKL) levels. Based on the previous studies and the present study, it can be argued that the combination of aerobic and resistance training (concurrent training) had the greatest effect on BMD and may be more beneficial than doing aerobics or resistance training alone to prevent osteoporosis in young people.

Calcium is an essential nutrient and important for bone health, peak bone mass, prevention and treatment of osteoporosis throughout life [38]. The main mechanisms involved in controlling bone regeneration and calcium homeostasis include changes in plasma PTH, calcitriol, calcium and phosphate, bone regeneration markers due to hypoparathyroidism and hyperparathyroidism, renal failure, daily PTH 1-34 administration, and RANKL inhibition [39]. Regarding the milk consumption and calcium rich resources, we can refer to Dionyssiotis et al. [40] study, who argued that physical activity and appropriate intake of calcium have beneficial effects on bone mass. They stated that women who did regular activity and calcium intake were more than 800 mg per day, had higher T-score than other groups who did not take calcium.

A study by Laird et al. [41] showed that high levels of yogurt are associated with an increase in BMD and exercise performance score. In addition, they first showed that with increasing consumption of yogurt, significantly decreased the odds of being characterized as osteopenic or osteoporotic in women and as osteoporotic in men. They linked this association to natural content of yogurt (yogurt naturally contains minerals and vitamins needed to develop bone density). Chen et al. [42] found that high calcium milk powder supplementation to delay and slow down bone loss was better than medium and low calcium supplementation in healthy postmenopausal women. A supplement containing 300 mg per day of calcium cannot reduce bone loss in the lumbar spine and greater Trochanter, but 600–900 mg per day can be beneficial. Also, Ikedo et al. [43] on Long-distance running women showed that taking vitamin D supplementation and low-fat milk for 6 months improved bone metabolism by maintaining the concentration of 25OH-D and decreasing concentration of PTH and reducing inflammatory cytokines. Furthermore, Rajatanavin et al. [38] stated that calcium supplementation of 500 mg per day can reduce bone turnover and decrease bone mass in older women.

Contrary to these studies, the meta-analysis by Tai et al. [44] suggested that increasing calcium intakes in daily food sources or by taking calcium supplements causes a slight increase in BMD and is unlikely to significantly reduce the risk of pancreatitis. Also, meta-analysis by Bolland et al. [45] indicated that the calcium intake is unrelated to bone fracture risk, and also argued that clinical evidence that increasing calcium intakes from food sources prevents fractures is weak and contradictory. The results of a study by Hirota et al. [46] demonstrated that lumbar spine BMD decreased after 4 months of diet compared to the initial value in both control and milk consumption groups. They suggested that muscle mass in milk consumption group was associated with changes in BMD of the lumbar spine and in subjects who had the greatest increase muscle mass, no reduction was observed in lumbar spine BMD.

Clinical trials have shown that calcium supplementation with or without vitamin D can reduce secondary hyperparathyroidism, slow down bone turnover, increase areal BMD, or slow down bone loss [6–8]. The beneficial effects of supplementation on bone density result from a decrease in bone remodeling space which lead to a decrease in the number of remodeling sites activated of bone, leading to reduction in bone resorption [47]. Calcium supplementation has an acute antiresorptive effect on bones which set bone reconstruction in a regular basis to maintain bone density or slow loss of the BMD, preserve cortical thickness by reducing endocortical resorption, and perhaps reduce the increase in intracortical porosity and/ or improve bone mineralization [47]. Based on the results of the previous and the present study, it seems that milk consumption (as a rich source of calcium) may have beneficial effects on bone mass of young people and prevent osteoporosis with aging. Moreover, combining milk consumption with concurrent training (aerobic-resistance) may increase its potential.

## Conclusions

The results of the present study showed that combining concurrent training with milk consumption on the BMD of young women with osteoporosis is more beneficial compared to separate milk consumption or training. Therefore, it seems that doing concurrent training and consuming milk can be used as an effective way to improve BMD in young women with diagnosed osteoporosis.

## Abbreviations

25OH-D: 25-hydroxyvitamin D
ALP: Alkaline phosphatase
BMD: Bone mineral density
BMI: Body mass index
C: Control
CT + MC: Concurrent training-milk consumption
CT: Concurrent training
MC: Milk consumption
CTX: Type 1 collagen C breakdown products
DEXA: Dual-energy x-ray absorptiometry
HRmax: Heart rate maximum
IOM: Institute of medicine
OPG: Osteoprotegerin
P1NP: Type 1 collagen amino-terminal propeptide
PTH: Parathyroid hormone
RANKL: Receptor activator of nuclear factor kappa B ligand
RM: Repetition maximum
WHO: World health organization
WHR: Waist to hip ratio
